# Diagnostic and prognostic utilities of multimarkers approach using procalcitonin, B-type natriuretic peptide, and neutrophil gelatinase-associated lipocalin in critically ill patients with suspected sepsis

**DOI:** 10.1186/1471-2334-14-224

**Published:** 2014-04-24

**Authors:** Mina Hur, Hanah Kim, Seungho Lee, Flavia Cristofano, Laura Magrini, Rossella Marino, Chiara Serena Gori, Cristina Bongiovanni, Benedetta Zancla, Patrizia Cardelli, Salvatore Di Somma

**Affiliations:** 1 Department of Laboratory Medicine, Konkuk University School of Medicine, Seoul, Korea; 2 School of Public Health, Seoul National University, Seoul, Korea; 3 Department of Medical-Surgery Sciences and Translational Medicine, School of Medicine and Psychology ‘Sapienza’ University of Rome, Emergency Medicine, Sant’ Andrea Hospital, Rome, Italy; 4 Department of Clinical and Molecular Medicine, School of Medicine and Psychology, ‘Sapienza’ University, Sant’Andrea Hospital, Rome, Italy

**Keywords:** B-type natriuretic peptide, Neutrophil gelatinase-associated lipocalin, Procalcitonin, Sepsis, Diagnosis, Prognosis

## Abstract

**Background:**

We investigated the diagnostic and prognostic utilities of procalcitonin (PCT), B-type natriuretic peptide (BNP), and neutrophil gelatinase-associated lipocalin (NGAL) in critically ill patients with suspected sepsis, for whom sepsis was diagnosed clinically or based on PCT concentrations.

**Methods:**

PCT, BNP, and NGAL concentrations were measured in 340 patients and were followed up in 109 patients. All studied biomarkers were analyzed according to the diagnosis, severity, and clinical outcomes of sepsis.

**Results:**

Clinical sepsis and PCT-based sepsis showed poor agreement (kappa = 0.2475). BNP and NGAL showed significant differences between the two groups of PCT-based sepsis (*P* = 0.0001 and *P* < 0.0001), although there was no difference between the two groups of clinical sepsis. BNP and NGAL were significantly different according to the PCT staging and sepsis-related organ failure assessment subscores (*P* < 0.0001, all). BNP and PCT concentrations were significantly higher in the non-survivors than in the survivors (*P* = 0.0002) and showed an equal ability to predict in-hospital mortality (*P* = 0.0001). In the survivors, the follow-up NGAL and PCT concentrations were significantly lower than the initial values (148.7 ng/mL vs. 214.5 ng/mL, *P* < 0.0001; 0.61 ng/mL vs. 5.56 ng/mL, *P* = 0.0012).

**Conclusions:**

PCT-based sepsis diagnosis seems to be more reliable and discriminating than clinical sepsis diagnosis. Multimarker approach using PCT, BNP, and NGAL would be useful for the diagnosis, staging, and prognosis prediction in the critically ill patients with suspected sepsis.

## Background

Sepsis is a systemic inflammatory response caused by infection. Progression to severe sepsis and septic shock is one of the main causes of high morbidity and mortality in critically ill patients in emergency or intensive care settings [1,2]. Since prompt and specific treatment in the patients with severe sepsis and septic shock has shown improved outcomes, it is crucial to ensure a timely diagnosis of this disease progression [3,4]. The sepsis-related organ failure assessment (SOFA) score has been used to describe and evaluate organ dysfunctions and failures in these patients [5].

Procalcitonin (PCT) has been extensively studied in various clinical settings, and growing evidences support its potential ability to diagnose sepsis, estimate its severity, and provide a prognosis [6-8]. Regarding organ dysfunctions and failures, various novel biomarkers have shown promise in clinical practice. B-type natriuretic peptide (BNP) has been used to successfully aid in the diagnosis of heart failure, and its concentrations correlated with both disease severity and prognosis [9-11]. Neutrophil gelatinase-associated lipocalin (NGAL) has been regarded as a sensitive, specific, and early predictive biomarker for acute kidney injury (AKI) [12-14]. In a recent meta-analysis, one of the major findings was that 41% of patients diagnosed with AKI would have been missed by creatinine alone [13].

A few recent studies reported a combined use of these novel biomarkers [15,16]. In a multicenter prospective study (GALLANT trial), the combination of plasma NGAL and BNP demonstrated clinical implications for risk stratification in the patients with acute heart failure, and plasma NGAL at discharge was a strong indicator of adverse outcomes [15]. Our previous study showed the diagnostic utility of plasma NGAL for AKI in the critically ill patients with suspected sepsis, for whom PCT was used for the diagnosis and staging of sepsis [16]. If several biomarkers, a marker for sepsis and respective markers for each organ dysfunction, are combined together, it will provide more objective and reliable guide for the diagnosis, risk stratification, prognosis, and treatment of sepsis. In the present study, we wanted to explore the diagnostic and prognostic utilities of PCT, BNP, and NGAL in critically ill patients with suspected sepsis, for whom sepsis was diagnosed clinically or based on PCT concentrations.

## Methods

### Study population

A prospective, observational study was conducted in two university hospitals: a 400-bed hospital (Sant’ Andrea Hospital, SAH) in Rome, Italy and a 900-bed hospital (Konkuk University Hospital, KUH) in Seoul, Korea. During the period from March 2012 to July 2013, a total of 340 patients (109 patients from SAH and 231 patients from KUH) were enrolled from the emergency department (n = 224) or intensive care unit (n = 116). They were critically ill patients with suspected sepsis, based on clinical diagnostic criteria of sepsis (e.g. SIRS) [17]. Their medical records were reviewed for the clinical and laboratory data. The protocol was designed following the criteria of the Declaration of Helsinki and was approved by the ethical committee of each participating hospital. The study protocol was approved by the Institutional Review Board (IRB) in each hospital (KUH and SAH). In KUH, written informed consent from the enrolled patients was exempted, because the biomarkers were measured using residual samples that would be discarded, without additional blood sampling from the patients. In SAH, written informed consent was obtained from all enrolled patients for the initial and follow-up data. The patients were divided into five groups (0 – 4) according to the cardiovascular, respiratory, and renal subscores of SOFA score [5]. Their median age was 67.5 years (range, 0 - 102 years), and 177 (52.1%) patients were males. The median duration of hospital stay was 10 days (range, 1 - 838 days), and in-hospital mortality was observed in 51 (15%) patients. Review of the medical history revealed cardiac diseases in 179 (52.6%) patients; diabetes in 90 (26.5%) patients; pulmonary diseases in 78 (22.9%) patients; cancers in 63 (18.5%) patients; gastric diseases in 46 (13.5%) patients; neurologic diseases in 45 (13.2%) patients; and chronic kidney diseases in 36 (10.6%) patients.

### Measurement of BNP, NGAL, and PCT

In the whole population, plasma BNP and NGAL were measured using the Triage CarioRenal Panel (Alere, Inc., San Diego, CA, USA), according to the manufacturer’s instruction [18]. It is a fluorescence immunoassay for the quantitative determination of BNP and NGAL in EDTA-anticoagulated whole blood and plasma specimens. Briefly, several drops of EDTA-anticoagulated whole blood are added to the sample port on the device within two hours after blood collection. Then, the blood cells are separated from the plasma using a filter contained in the device. The specimen reacts with fluorescent antibody conjugates and flows through the device by capillary action. Complexes of each fluorescent conjugate are captured on a discrete zone specific for each analyte. The concentration of each analyte is directly proportional to the fluorescence detected in the Triage Meter (Alere, Inc.) and is displayed approximately 20 mins later. The medical decision points were regarded as 100 pg/mL and 150 ng/mL for plasma BNP and NGAL, respectively [13,15].

Serum PCT was measured using the Elecsys BRAHMS PCT electrochemiluminescence assay (BRAHMS, Henningsdorf, Germany) in the Roche Cobas e-System (Roche Diagnostics, Basel, Switzerland) or BRAHMS PCT sensitive Kryptor immunofluorescent assay system (BRAHMS). Both are fully automated, sensitive, quantitative PCT assays with functional sensitivity of 0.06 ng/mL. On the basis of the previous studies and manufacturers’ recommendation, PCT concentrations were divided into five groups: < 0.05 ng/mL, group I (healthy); 0.05 – 0.49 ng/mL, group II (local infection); 0.5 – 1.99 ng/mL, group III (systemic infection or sepsis); 2.0 – 9.99 ng/mL, group IV (severe sepsis); and ≥ 10 ng/mL, group V (septic shock) [16,19]. Table 1 shows the distribution of study population according to the PCT concentrations.

**Table 1:** Distribution of study population according to the procalcitonin concentrations

**PCT (ng/mL)**	**Interpretation**	**n**	**Blood culture positive (%)**	**Age (yr)**	**Male (%)**
I (<0.05)	Healthy	28	1 (3.6)	68 (5 - 89)	13 (46.4)
II (0.05 – 0.49)	Local infection	162	10 (6.2)	67 (0 - 100)	90 (55.2)
III (0.5 – 1.99)	Systemic infection or sepsis	75	9 (12.0)	65.5 (0 - 96)	31 (41.9)
IV (2.0 – 9.99)	Severe sepsis	42	9 (21.4)	70 (0 - 95)	20 (47.6)
V (≥10)	Septic shock	33	11 (33.3)	70 (46 - 102)	23 (69.7)
Total		340	40 (11.8)	67.5 (0 - 102)	177 (52.1)

Age is expressed as median value (range).

Blood culture-positive rates tended to increase according to the PCT groups, showing significant differences (I vs. V, *P* = 0.0097;

II vs. IV, *P* = 0.0065; II vs. V, *P* < 0.0001; III vs. V, *P* = 0.0184).

*PCT*: procalcitonin.

The samples for BNP, NGAL, and PCT were obtained simultaneously, when the patients showed acutely ill-looking appearances with the changes of vital signs and were suspected of having sepsis [17]. In 109 of all cohort of 340 patients, NGAL and PCT concentrations were also followed up after 72 hours of initial measurement.

### Statistical analysis

Chi-square test was used to compare the proportions. Mann-Whitney test was used to compare BNP, NGAL, and creatinine according to the sepsis diagnosis and to compare BNP, NGAL, and PCT according to the in-hospital mortality. Agreement between the diagnosis of clinical sepsis and PCT-based sepsis was assessed using Cohen’s Kappa (agreement: < 0.4, poor; 0.4 – 0.75, fair to good; > 0.75, excellent). Kruskal-Wallis test was used to compare BNP, NGAL, and creatinine according to the PCT groups and to compare the distribution of NGAL and BNP according to the subscores of SOFA score. Paired t-test was used to compare NGAL and PCT at initial measurement and follow-up according to the in-hospital mortality. Receiver operating characteristic (ROC) curves were analyzed to compare BNP, NGAL, and PCT in predicting in-hospital mortality and in diagnosing sepsis. Logistic regression was also used to compare NGAL and PCT in predicting in-hospital mortality. For the statistical analyses, SPSS software (version 17.0, SPSS Inc., Chicago, IL, USA) and MedCalc software (version 12.1.4, MedCalc Software, Mariakerke, Belgium) were used. *P* values equal to or less than 0.05 were considered statistically significant.

## Results

According to the PCT concentrations, 44.1% (150/340) of the patients belonged to the groups of sepsis, severe sepsis, or septic shock (Table 1). Blood culture-positive rates increased significantly according to the PCT groups. When sepsis was diagnosed clinically or based on the PCT concentrations, the overall concordance rate between the two diagnoses was 63.8% (217/340), showing a poor agreement (kappa = 0.2425) (Table 2). In spite of this poor agreement, the positive rate of blood culture in the patients with clinical sepsis (21.0%, 25/119) was not different from that in the patients with PCT-based sepsis (19.3%, 29/150), showing a difference of 1.70 (95% confidence interval [CI], -8.32% to 12.03%, *P* = 0.8473).

**Table 2:** **Comparison between clinical sepsis and procalcitonin**-**based sepsis**

	**PCT-based sepsis**		**Kappa (95% CI)**
	**Negative (n = 190)**	**Positive (n = 150)**	
Clinical sepsis			
Negative (n = 221)	144	77	
Positive (n = 119)	46	73	
			0.2425 (0.1398 – 0.3453)

*P* = 0.0071 (McNemar test).

The critically ill patients with suspected sepsis were diagnosed on the basis of clinical diagnostic criteria of sepsis (e.g. SIRS) [17].

*PCT*: procalcitonin; *CI*: confidence interval.

The concentrations of BNP, NGAL, and creatinine were compared according to the sepsis diagnosis (Table 3). Although, according to the clinical diagnosis of sepsis, there was no statistical difference in both BNP and NGAL concentrations, these two biomarkers significantly differed between the two groups of PCT-based sepsis. Such a difference was also observed in the comparison of creatinine. For the clinical diagnosis of sepsis, the area under the ROC curve (AUC with 95% CI) for PCT (0.673 [0.621 – 0.723]) differed significantly from those of BNP (0.526 [0.472 – 0.580], *P* < 0.001) and NGAL (0.509 [0.455 – 0.564], *P* = 0.005). For the PCT-based diagnosis of sepsis, both BNP and NGAL showed an ability to distinguish between the two groups of sepsis. The AUCs (95% CI) for BNP and NGAL were 0.634 (0.580 – 0.685) and 0.594 (0.539 – 0.646), respectively, showing no statistical difference between the two AUCs (*P* = 0.426).

**Table 3:** **Comparison of NGAL**, **creatinine**, **and BNP according to the sepsis diagnosis**

	**Clinical sepsis (n = 119)**		**PCT**-**based sepsis (n = 150)**	
	**Non**-**sepsis (n = 221)**	**Sepsis (n = 119)**	**Non**-**sepsis (n = 190)**	**Sepsis (n = 150)**
BNP (pg/mL)	72.3 (52.8 – 115.7)	140 (65.5 – 189.3)	66.7 (30.8 – 96.0)	146.5 (93.5 – 223.4)^*^
NGAL (ng/mL)	184 (161.9 – 233.6)	210 (137.0 – 265.0)	137 (115.0 – 167.0)	333 (255.6 – 448.0)^†^
Creatinine (mg/dL)	0.94 (0.85 – 1.09)	0.84 (0.71 – 1.10)	0.81 (0.76 – 0.93)	1.12 (0.98 – 1.50)^†^

^*^P = 0.0001 vs. non-sepsis.

^†^*P* < 0.0001 vs. non-sepsis.

Data are expressed as median (95% confidence interval).

*BNP*: *B*-type natriuretic peptide; *NGAL*: neutrophil gelatinase-associated lipocalin; *PCT*: procalcitonin.

**Figure 1:**
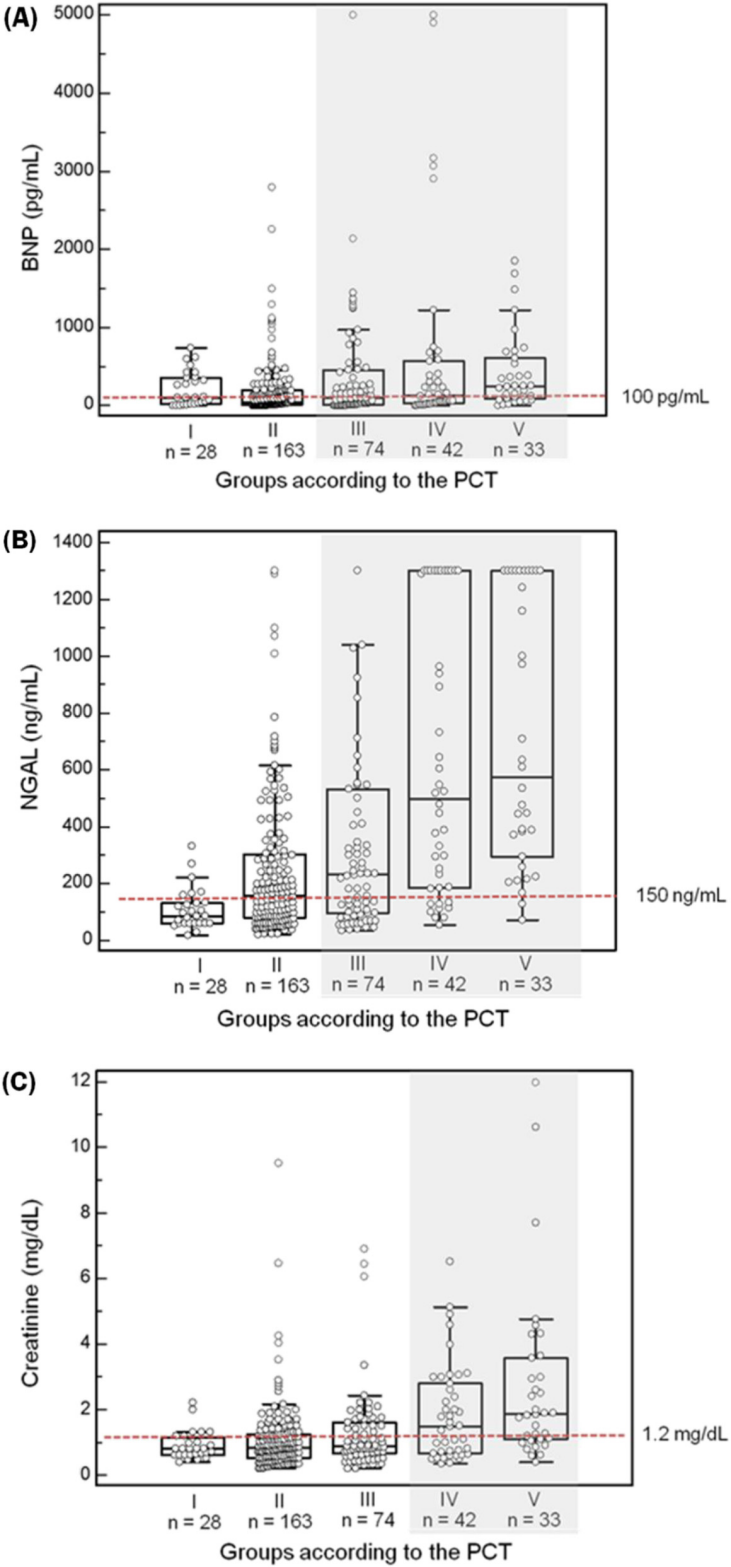
**Comparison of BNP, NGAL, and creatinine according to the PCT groups. (A)** The BNP concentrations according to the PCT groups were: 109 (5 – 1,220) pg/mL; 42.2 (5 – 2,790) pg/mL; 103.5 (5 – 5,000) pg/mL; 129 (5 – 5,000) pg/mL; and 241 (5 – 1,850) pg/mL, respectively (*P* = 0.0001). The median values of BNP were all above the medical decision point (100 pg/mL) in septic patients. **(B)** The NGAL concentrations according to the PCT groups were: 90.5 (16 – 444) ng/mL; 156 (19 – 1,300) ng/mL; 232 (33 – 1,301) ng/mL; 499.5 (55 – 1,301) ng/mL; and 609 (72 – 1,300) ng/mL, respectively (*P* < 0.0001). The median values of NGAL were all above the medical decision point (150 ng/mL) in septic patients. **(C)** The creatinine concentrations according to the PCT groups were: 0.8 (0.39 – 11.98) mg/dL; 0.82 (0.2 – 9.52) mg/dL; 0.875 (0.2 – 6.9) mg/dL; 1.49 (0.33 – 6.52) mg/dL; and 1.85 (0.4 – 10.61) mg/dL, respectively (*P* < 0.0001). The median value of creatinine was above the medical decision point (1.2 mg/dL) only in severe sepsis & septic shock patients.

**Figure 2:**
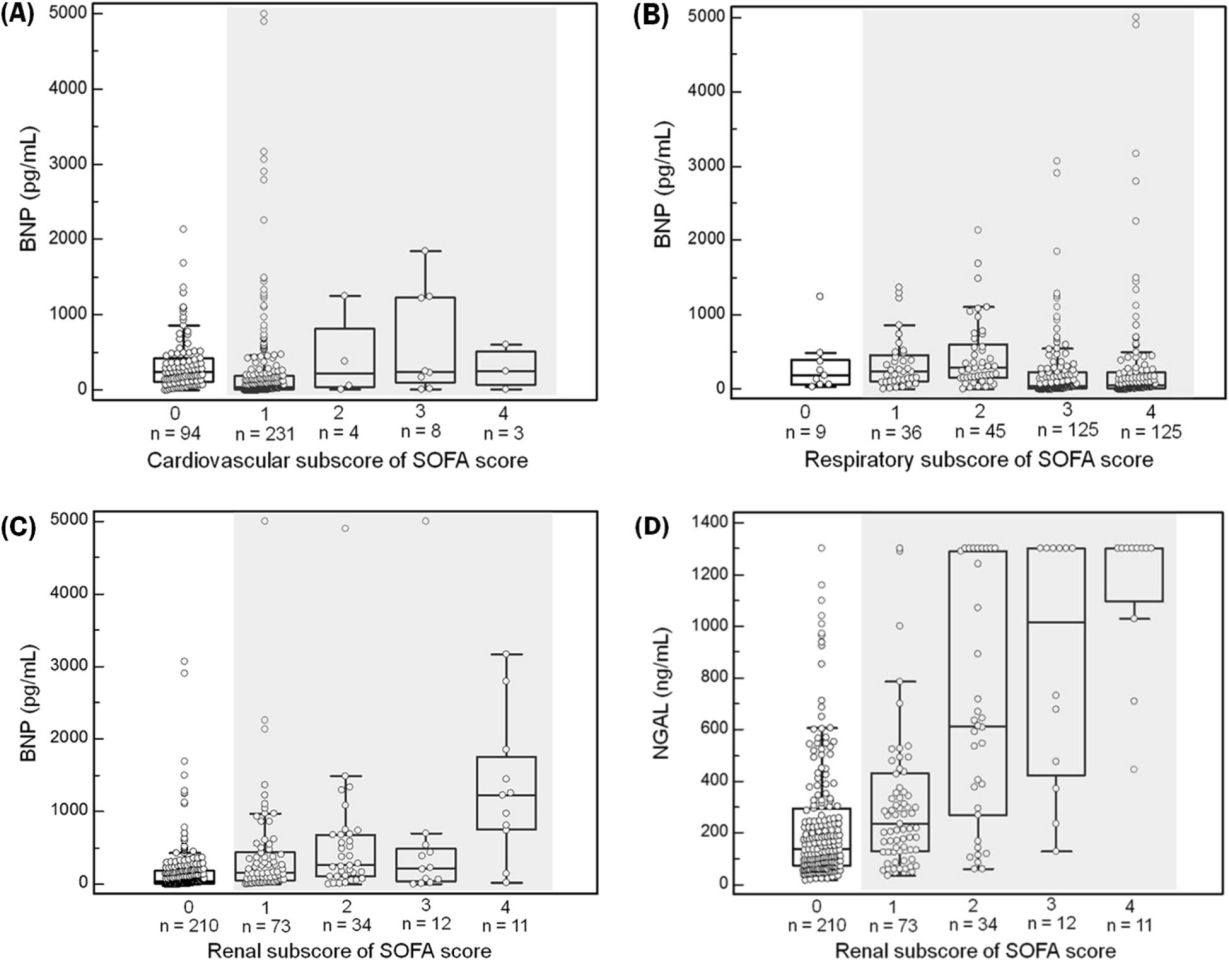
**Comparison of BNP and NGAL according to the subscores of SOFA score. (A)** The BNP concentrations according to the cardiovascular subscore of SOFA score were: 245.5 (5 – 2,140) pg/mL; 43 (1 – 5,000) pg/mL; 223.9 (12.6 – 1,260) pg/mL; 245 (13.5 – 1,850) pg/mL; and 257 (10.1 – 609) pg/mL, respectively. The BNP concentration was significantly associated with the cardiovascular subscore of SOFA score (*P* < 0.0001). **(B)** The BNP concentrations according to the respiratory subscore of SOFA score were: 180 (35 – 1,240) pg/mL; 232.5 (5 – 1,370) pg/mL; 281 (5 – 2,140) pg/mL; 43 (5 – 3,070) pg/mL; and 46.6 (5 – 5,000) pg/mL, respectively. Although the BNP concentration was significantly different according to the respiratory subscore of SOFA score, there was no increasing tendency (*P* < 0.0001). **(C)** The BNP concentrations according to the renal subscore of SOFA score were: 43.7 (5 – 3,070) pg/mL; 147 (5 – 5,000) pg/mL; 266.5 (5 – 4,900) pg/mL; 211.5 (5 – 5,000) pg/mL; and 1,220 (19.4 – 3,170) pg/mL, respectively. The BNP concentration was significantly associated with the renal subscore of SOFA score (*P* < 0.0001). **(D)** The NGAL concentrations according to the renal subscore of SOFA score were: 137 (16 – 1,301) ng/mL; 234 (33 – 1,300) ng/mL; 612 (59 – 1,301) ng/mL; 1015.5 (127 – 1,300) ng/mL; and 1,300 (444 – 1,300) ng/mL, respectively. The NGAL concentration was significantly associated with the renal subscore of SOFA score (*P* < 0.0001). Abbreviations: BNP, B-type natriuretic peptide; NGAL, neutrophil gelatinase-associated lipocalin; SOFA, sepsis-related organ failure assessment.

BNP, NGAL, and creatinine all showed significant differences according to the PCT groups (*P* < 0.0001 for NGAL and creatinine; *P* = 0.0001 for BNP) (Figure 1). The median concentrations of BNP and NGAL were above the medical decision points (100 pg/mL and 150 ng/mL, respectively) in septic patients, while that of creatinine was above the medical decision point (1.2 mg/dL) only in severe sepsis & septic shock patients. The distributions of BNP and NGAL according to the subscores of SOFA score are presented in Figure 2. Plasma BNP concentration was significantly associated with the cardiovascular and renal subscores of SOFA score (*P* < 0.0001). Although plasma BNP concentration differed significantly according to the respiratory subscore of SOFA score, there was no increasing tendency. Plasma NGAL concentration was significantly associated with the renal subscore of SOFA score (*P* < 0.0001).

BNP, NGAL, and PCT concentrations were analyzed according to the in-hospital mortality (Table 4). In a total of 340 patients, BNP and PCT concentrations were significantly higher in the non-survivors than in the survivors (*P* = 0.0002, both). There was no statistical difference in NGAL concentrations between the two groups (*P* = 0.1804). Regardless of clinical sepsis or PCT-based sepsis, BNP concentrations were significantly higher in the non-survivors than in the survivors (*P* = 0.0014 and *P* = 0.0054). In the ROC curve analysis, both BNP & PCT showed an equal ability to predict in-hospital mortality: the AUC for BNP and PTC was 0.661 (95% CI, 0.608 – 0.711; *P* = 0.0001).

**Table 4:** **Comparison of BNP**, **NGAL**, **and PCT according to the in**-**hospital mortality**

	**Total (n = 340)**		**Clinical sepsis (n = 119)**		**PCT**-**based sepsis (n = 150)**	
	**Survivors (n = 288)**	**Non**-**survivors (n = 51)**	**Survivors (n = 82)**	**Non**-**survivors (n = 37)**	**Survivors (n = 117)**	**Non**-**survivors (n = 33)**
BNP (pg/mL)	69 (46.7 – 101.0)	200^*^ (159.1 – 280.2)	48.2 (13.4 – 143.1)	237.0^†^ (165.2 – 356.7)	112.0 (63.8 – 157.5)	253.0^‡^ (181.2 – 519.2)
NGAL (ng/mL)	184 (159.1 – 222.7)	227 (161.4 – 444.9)	189 (108.6 – 304.7)	218 (143.5 – 375.1)	346 (281.9 – 490.6)	254 (145.5 – 519.5)
PCT (ng/mL)	0.27 (0.23 – 0.39)	0.93^*^ (0.54 – 1.35)	0.76 (0.43 – 1.52)	0.93 (0.63 – 1.34)	NA	

^*^P = 0.0002 vs. survivors.

^†^*P* = 0.0014 vs. survivors.

^‡^*P* = 0.0054 vs. survivors.

Data are expressed as median (95% confidence interval).

*BNP*: B-type natriuretic peptide; *NGAL*: neutrophil gelatinase-associated lipocalin; *PCT*: procalcitonin; *NA*: not applicable.

The initial and follow-up concentrations of NGAL and PCT were further compared according to the in-hospital mortality (Table 5). In the survivors, the follow-up NGAL and PCT concentrations were significantly lower than their initial concentrations (*P* < 0.0001 and *P* = 0.0012, respectively). In logistic regression, they contributed significantly to the prediction of in-hospital mortality. Odds ratios (95% CI) were 1.0032 (1.0012 – 1.0052) and 1.8485 (1.2742 – 2.6815) in NGAL and PCT, respectively.

**Table 5:** **Comparison of NGAL and PCT at initial and follow**-**up measurements according to the in**-**hospital mortality**

	**Survivors (n = 96)**		**Non**-**survivors (n = 13)**	
	**Initial measurement (mean ± SD)**	**Follow-up measurement (mean ± SD)**	**Initial measurement (mean ± SD)**	**Follow-up measurement (mean ± SD)**
NGAL (ng/mL)	214.5 ± 249.1	148.7 ± 173.3^*^	727.4 ± 554.5	775.7 ± 562.1
PCT (ng/mL)	5.56 ± 15.4	0.61 ± 2.18^†^	31.8 ± 72.6	18.4 ± 37.4

^*^P < 0.0001 vs. initial measurement.

^†^*P* = 0.0012 vs. initial measurement.

Both initial and follow-up data were available in 109 patients (96 survivors and 13 non-survivors).

*NGAL*: neutrophil gelatinase-associated lipocalin; *PCT*: procalcitonin; *SD*: standard deviation.

## Discussion

The potential clinical usefulness of some innovative biomarkers has been discussed in the diagnosis, staging, and monitoring of sepsis, and these biomarker-guided strategies may allow more refined risk stratification and lead to improved patient care and outcomes [20,21]. To the best of our knowledge, however, there have been no studies that investigated the three biomarkers of BNP, NGAL, and PCT together in the septic patients so far.

In the present study, we investigated the diagnostic and prognostic utilities of BNP, NGAL, and PCT in critically ill patients with suspected sepsis. In particular, we diagnosed and graded the sepsis based on the PCT concentrations, in addition to the clinical diagnosis. Noticeably, there was a poor agreement between the clinical diagnosis and PCT-based diagnosis of sepsis (Table 2). In spite of this poor agreement, the positive rate of blood culture in the patients with clinical sepsis did not differ from that in the patients with PCT-based sepsis (21.0% vs. 19.3%), and the blood culture-positive rates increased significantly according to the PCT groups (Table 1). Eleven out of 40 patients with positive blood culture belonged to PCT groups I or II. Despite being considered the gold standard for diagnosis, blood cultures are subject to contamination by skin flora if blood cultures are not collected using an appropriate aseptic technique [22]. In this regard, it is possible that the positive blood culture results might have been due to contamination. In recent studies, initial PCT concentrations in the emergency department accurately predicted blood culture positivity in patients with community-acquired pneumonia [23], and PCT showed excellent correlation with bacterial load and could discriminate between blood culture contaminants and organisms representing bacteremia [24].

Similar to our data, a multicenter study reported a large disconnect between the perceived severity of heart failure (as assessed by initial disposition and New York Heart Association functional class) and the BNP level, emphasizing the biomarker-guided therapeutic and monitoring strategies involving BNP in the patients with heart failure [9]. Another interesting finding is that the BNP and NGAL concentrations demonstrated significant differences according to the presence or absence of sepsis only when they were compared according to the PCT-based sepsis (Table 3). Based on these results, PCT-based sepsis diagnosis seems to be more reliable and discriminating than clinical sepsis diagnosis.

The assessment of risk stratification and prognosis in sepsis is based on the clinical scoring systems. However, they are in general inefficient to provide definite clues on organ dysfunctions or failures [5,20]. In this study, BNP, NGAL, and creatinine all showed significant differences according to the PCT groups (Figure 1). Although creatinine was increased only in the patients with severe sepsis & septic shock, BNP and NGAL were increased above their medical decision points in all septic patients. Compared with creatinine, NGAL seems to be an early, objective marker of AKI in relation to sepsis [16]. Moreover, the BNP concentration was significantly associated with the cardiovascular and renal subscores of SOFA score, and the NGAL concentration was significantly associated with the renal subscore of SOFA score (Figure 2). Taken together, these findings imply the clinical usefulness of these innovative biomarkers to assess the organ dysfunctions or failures along with the sepsis severity.

We also observed the significant differences of these biomarkers according to the in-hospital mortality (Table 4). The initial BNP and PCT concentrations were significantly higher in the non-survivors than in the survivors, and both BNP & PCT showed an equal ability to predict in-hospital mortality. In the survivors, the follow-up NGAL and PCT concentrations were significantly lower than their initial concentrations (Table 5). The present findings support the previous studies, which showed the usefulness of these biomarkers for prognostic stratification in various critical care settings [15,25,26]. PCT seems to be associated with the diagnosis and severity of sepsis, BNP with response to circulatory and cardiovascular complications, and NGAL with AKI in severe sepsis/septic shock. Compared with PCT and BNP, NGAL did not seem to correlate with mortality. This study is partly limited in that the follow-up data of biomarkers were available only in one thirds of the enrolled patients and included only NGAL and PCT. Accordingly, it is difficult to draw any conclusions from these results, and serial measurement of these biomarkers would be valuable to estimate the adverse outcomes in the patients with sepsis.

## Conclusions

In conclusion, this is the first study that investigated the combined use of BNP, NGAL, and PCT in critically ill patients with suspected sepsis. A substantial discrepancy may exist between the clinical diagnosis and PCT-based diagnosis of sepsis, and PCT-based sepsis diagnosis seems to be more reliable and discriminating than the clinical diagnosis. Multimarker strategy including BNP, NGAL, and PCT seems to be an objective and useful approach for the diagnosis, staging, and prognosis prediction in the critically ill patients with suspected sepsis. Further studies are warranted to facilitate and refine the combined use of biomarkers in a variety of critical care settings.

## Abbreviations

PCT: Procalcitonin; BNP: B-type natriuretic peptide; NGAL: Neutrophil gelatinase-associated lipocalin; SAH: Sant’Andrea hospital; KUH: Konkuk university hospital; SOFA: Sepsis-related organ failure assessment; AKI: Acute kidney injury; ROC: Receiver operating characteristic; AUC: Area under the curve; CI: Confidence interval.

## Competing interests

The authors declare that they have no competing interests.

## Authors’ contributions

MH designed the study and participated in elaboration of data, in writing the manuscript; HK participated in the enrollment of the patients, elaborating data, statistical analysis, and in writing manuscript; SL participated in elaborating data, in statistical analysis, and in writing manuscript; FC participated in the enrollment of the patients; LM participated in the design of the study, in the enrollment of the patients, elaboration of data, and in revising manuscript; RM participated in the enrollment of the patients; CSG participated in the enrollment of the patients and in the elaboration of data; CB participated in the enrollment of the patients; BZ participated in the enrollment of the patients; PC participated in carrying out the assays; SDS participated in the design of the study, elaboration of data, and revised the manuscript. All authors read and approved the final manuscript.

## Pre-publication history

The pre-publication history for this paper can be accessed here:

http://www.biomedcentral.com/1471-2334/14/224/prepub
